# Supplementary material for: Sparse dimensionality reduction for analyzing single-cell-resolved interactions
Source: Bioinform Adv. 2026 Mar 25;6(1):vbag047. doi: 10.1093/bioadv/vbag047 (PMC13014469; doi:10.1093/bioadv/vbag047)
Supplement: vbag047_Supplementary_Data [file vbag047_supplementary_data.pdf]

Supplementary material for Sparse dimensionality reduction for analyzing single-cell-resolved interactions

Niklas Brunn[1,2,*], Maren Hackenberg[1,2], Camila L. Fullio[3,4],
Tanja Vogel[3], Harald Binder[1,2,5]

[1] Institute of Medical Biometry and Statistics (IMBI), Faculty of Medicine and Medical Center, University of Freiburg, Germany
[2] Freiburg Center for Data Analysis, Modeling and AI (FDMAI), University of Freiburg, Germany
[3] Institute of Anatomy and Cell Biology, Department Molecular Embryology, Faculty of Medicine, University of Freiburg, Germany
[4] Faculty of Biology, University of Freiburg, Germany
[5] Centre for Integrative Biological Signaling Studies (CIBSS), University of Freiburg, Germany

[*] *Corresponding author:* `niklas.brunn@uniklinik-freiburg.de`

Contents

S1 Boosting autoencoder 2
- S1.1 Boosting autoencoder optimization algorithm . . . 2
- S1.2 Boosting autoencoder architecture . . . 2
- S1.3 Boosting autoencoder training . . . 2
- S1.4 Boosting autoencoder sparsity control . . . 3
- S1.5 Boosting autoencoder implementation . . . 4
- S1.6 Boosting autoencoder functionality . . . 4

S2 Sparsity of interaction patterns 4

S3 Cell-cell interaction data preprocessing 4
- S3.1 Preprocessing of raw gene expression data . . . 5
- S3.2 NICHES cell-cell interaction matrix construction algorithm . . . 5
- S3.3 Preprocessing of the cell-cell interaction matrix . . . 6

S4 Boosting autoencoder scalability analysis 6

S5 Analysis of BAE-identified interactions in rat lung scRNA-seq data 10

S6 Analysis of anterior mouse brain spatial transcriptomics data 10

S7 References 18

S1 Boosting autoencoder

S1.1 Boosting autoencoder optimization algorithm

Compared to the original publication of the BAE (Hackenberg et al., 2025), we optimized the implementation of the approach to speed up the computations. First, we applied weight decay to the decoder parameters as proposed in Loshchilov et al., 2017. Regularizing the decoder parameters during optimization prevents the model from overfitting even if training is conducted for a larger number of epochs. Second, we now compute the gradients required for updating the parameters of all model components simultaneously, thereby speeding up the optimization process. Third, instead of only selecting and updating the optimal encoder weight for each latent dimension in each componentwise boosting iteration, we now allow all previously selected weights to be updated together with the current selected weight. This provides more flexibility to the boosting model, which attempts to iteratively fit changing responses based on the negative gradients of the autoencoder reconstruction loss w.r.t. the latent representation throughout the optimization process. Finally, a soft clustering component can be integrated into the modeling framework by adding a $\mathrm{split\text{-}softmax}$ transformation after the encoder, as explained in the main text. Formally, for n observations, let $\boldsymbol{Z} \in \mathbb{R}^{d \times n}$ denote the output of the encoder which is constructed by multiplying the data matrix $\boldsymbol{X} \in \mathbb{R}^{p \times n}$ with the sparse encoder weight matrix $\boldsymbol{W} \in \mathbb{R}^{d \times p}$, where $d \ll p$. The $\mathrm{split\text{-}softmax}$ transformation first splits the activation in latent dimensions per cell pair into their positive and negative version, followed by a $\mathrm{softmax}$ transformation $\sigma : \mathbb{R}^d \to \mathbb{R}^d$, to model associations of cell pairs to different latent dimensions. This can be formalized by the mapping

$$\begin{aligned} &\sigma_{\mathrm{split}} : \mathbb{R}^d \to \mathbb{R}^{2d} \\ &(z_1, \ldots, z_d)^\top \mapsto \sigma((z_1, -z_1, \ldots, z_d, -z_d)^\top). \end{aligned}$$

S1.2 Boosting autoencoder architecture

We used the following BAE architecture for analyzing the CCIM constructed from the rat lung scRNA-seq data (Raredon et al., 2019) by applying NICHES (Raredon et al., 2023). The encoder was defined to consist of one linear layer $f_{\mathrm{enc}} : \mathbb{R}^p \to \mathbb{R}^d$, i.e., without a bias vector. The decoder consists of three layers of which two have trainable parameters. The first layer of the decoder is the $\mathrm{split\text{-}softmax}$ transformation $\sigma_{\mathrm{split}} : \mathbb{R}^d \to \mathbb{R}^{2d}$ without trainable parameters, followed by a fully connected layer with $\tanh$ activation $f_{\mathrm{dec}_1} : \mathbb{R}^{2d} \to \mathbb{R}^p$. The last layer was defined to be a fully connected affine layer $f_{\mathrm{dec}_2} : \mathbb{R}^p \to \mathbb{R}^p$.

For the CCIM computed by NICHES using the subset of the rat lung data (Raredon et al., 2019), the number of ligand-receptor interactions detected by NICHES was $p = 1740$ and for the BAE analysis we set $d = 30$, such that cell pairs could be assigned to 60 different clusters. An exemplary model overview figure is provided in the main text in Fig. 1. a.

S1.3 Boosting autoencoder training

Prior to training, the BAE works best if the input features are standardized across the observations, i.e., z-scores of each feature vector were computed and used as the input for training the model. Each feature vector can either correspond to a detected ligand-receptor interaction by NICHES, consisting of the interaction scores of individual cell pairs, or to gene expressions of cells in a gene when analyzing single-cell gene expression data.

For training the model, decoder parameters were randomly initialized using the Xavier initialization strategy (Glorot et al., 2010) and the encoder weights were set to zero for learning sparse connections.

We choose the mean squared error as the model reconstruction loss since the input data was standardized and used a well known modification of the Adam optimizer (Kingma et al., 2014), AdamW (Loshchilov et al., 2017), for optimizing the decoder parameters. The AdamW optimizer makes $L2$ regularization equivalent to weight decay, which is not the case for the vanilla Adam optimizer when adding a $L2$ penalty term for the decoder parameters to the reconstruction loss.

The BAE was trained for 2000 epochs, where in each epoch, the input data was randomly divided into mini-batches of size 2^{12} and each mini-batch was used to update the parameters during each epoch.

The BAE optimization framework, consisting of a boosting component and the AdamW optimizer, makes use of gradient feedback, which is computed using the forward pass of the model with the current state of all model parameters using one mini-batch respectively. Negative gradients of the model loss with respect to the decoder parameters were then further passed to the AdamW optimizer, whereas negative gradients with respect to the latent representation of samples in the mini-batch in different latent dimensions were handed over to the componentwise boosting component for sparse feature selection and disentanglement of latent dimensions (Hackenberg et al., 2025).

We set the learning rate for the AdamW optimizer to 0.01, the weight decay parameter to 0.1, the decay parameter for the first and the second momentum estimate to the default values, and the step size for the boosting component to 0.001. We limited the number of boosting steps performed during each parameter update iteration for each latent dimension to 1 since componentwise boosting is integrated into an iterative optimization scheme. More generally, we found that these values depict suitable candidates for default values for training a BAE, except for the batch size and the number of training epochs, which can be chosen based on the number of observations in the data and control the sparsity level of the resulting encoder weight matrix (see also S1.4).

S1.4 Boosting autoencoder sparsity control

During training, a BAE model learns a sparse weight matrix $\boldsymbol{W} \in \mathbb{R}^{d \times p}$ that maps p input features, such as ligand-receptor interactions, onto d latent dimensions. The sparsity of $\boldsymbol{W}$ is governed by specific model and training hyperparameters (see also Hackenberg et al., 2025, Methods Section).

The number of nonzero weights after training is bounded by the number of boosting steps per epoch, M, and the number of training epochs, N. If the encoder weights are updated once per epoch, the total number of selected features $k \in \mathbb{N}$ lies within the range:

$$d \leq k \leq dMN,$$

with the number of features per individual latent dimension satisfying:

$$1 \leq k \leq MN.$$

Standard deep learning libraries such as Flux (Julia, our implementation) or PyTorch (Python) typically perform parameter updates over batches that partition the training data. Consequently, the batch size B, with $1 \leq B \leq n$ (where n is the number of observations in the data), must also be considered when estimating the number of nonzero weights. In such cases, the bounds adjust to:

$$d \leq k \leq dNM\lceil n/B \rceil,$$

and for individual latent dimensions:

$$1 \leq k \leq NM\lceil n/B \rceil.$$

S1.5 Boosting autoencoder implementation

The BAE was implemented using the Julia programming language (Bezanson et al., 2017), v1.9.3 (macOS, aarch64). We used the Flux (v0.14.15) library for deep learning models (Innes, 2018) which is based on the Zygote framework (Innes et al., 2019) for automatic differentiation. Other Julia packages required for the implementation are CSV v0.10.14, Clustering v0.15.7, ColorSchemes v3.25.0, DataFrames v1.6.1, Distances v0.10.11, IJulia v1.25.0, Plots v1.40.4, ProgressMeter v1.10.0, RCall v0.14.1, StatsBase v0.34.3, UMAP v0.1.11, VegaLite v3.3.0. All experiments were conducted on a MacBook Pro (2023) with an Apple M2 Max chip (12-core CPU, 38-core GPU) and 96 GB of RAM.

S1.6 Boosting autoencoder functionality

To demonstrate the functionality of the BAE with the disentanglement constraint, especially to highlight the capabilities of the soft clustering component, we added a tutorial notebook in our GitHub repository https://github.com/NiklasBrunn/Sparse-dimension-reduction. In this tutorial notebook users can run an exemplary BAE analysis on simulated binary scRNA-seq count data oriented on the simulation design proposed in Hess et al., 2020. In this case, the observations correspond to cells and the features correspond to genes.

S2 Sparsity of interaction patterns

While biological signaling networks are not inherently sparse, gene expression data derived from single-cell RNA sequencing is typically both noisy and sparse, largely due to technical limitations (e.g., dropout effects) and biological variability (Andrews et al., 2018). When constructing cell-cell interaction matrices (CCIMs), this sparsity is further amplified. Specifically, CCIMs consist of ligand-receptor coexpression scores, calculated as the product or geometric mean of ligand expression in sender cells and receptor expression in receiver cells (e.g. Raredon et al., 2023; Wilk et al., 2024). Hence, the multiplicative combination can lead to even sparser interaction matrices.

This high level of sparsity poses a challenge for models that aim to fully reconstruct the full interaction space. However, it is well suited to approaches that focus on identifying key interaction patterns. The Boosting Autoencoder (BAE) takes advantage of this sparsity by learning concise and interpretable representations that emphasize the most informative ligand-receptor interactions, rather than attempting to model all possible interactions. This trade-off allows the model to concentrate on biologically meaningful signals while reducing the influence of noise.

S3 Cell-cell interaction data preprocessing

The input to the BAE dimensionality reduction model is a cell-cell interaction matrix (CCIM), where each row represents a directed interaction between a sender and a receiver cell, and each column corresponds to an expression-based score for a specific ligand-receptor pair. This matrix serves

as a proxy for the cell-cell communication landscape at single-cell resolution. The BAE uses this representation to learn latent interaction patterns, each defined by a small, interpretable set of model-selected ligand-receptor interactions that are shared across clusters of cell pairs and reflect biologically meaningful signaling events.

In the following sections, we provide a description of the preprocessing steps, starting from raw gene expression data and leading to the construction of the preprocessed CCIM using NICHES (Raredon et al., 2023).

S3.1 Preprocessing of raw gene expression data

Raw gene expression data from single-cell RNA sequencing (scRNA-seq) or spatial transcriptomics experiments should undergo assay-specific quality control procedures as a first step (e.g. Luecken et al., 2019). These typically include the removal of low-quality cells or spots (e.g., based on mitochondrial gene content or total UMI counts) and lowly expressed genes. After filtering, the resulting count matrix can be log-normalized by computing relative counts (counts per gene divided by the total counts per cell), multiplying by a scale factor (e.g., 10,000), and applying the natural logarithm with a pseudocount of 1:

$$\text{log-normalized expression} = \log\left(\frac{\text{counts} \cdot 10000}{\text{total counts per cell}} + 1\right)$$

If the NICHES framework is used to construct a CCIM from scRNA-seq data, cell type annotations are required in advance. In that case, NICHES performs interaction inference based on ligand-receptor coexpression and uses a subsampling strategy to manage computational scalability, as the number of possible cell-cell interaction pairs increases quadratically with the number of cells (Raredon et al., 2023).

S3.2 NICHES cell-cell interaction matrix construction algorithm

For a comprehensive description of the NICHES algorithm, including its method for constructing cell-cell interaction matrices (CCIMs), underlying assumptions, limitations, and usage guidelines, we refer readers to the Supplementary Material of the original NICHES publication (Raredon et al., 2023). Below, we provide a brief overview of the CCIM construction process as implemented in the NICHES software.

NICHES (Niche Interactions and Communication Heterogeneity in Extracellular Signaling) is an R-based software package for characterizing cellular interactions at the single-cell level using ligand-receptor expression profiles. It also supports the construction of low-dimensional embeddings of interaction data (Raredon et al., 2023).

The algorithm takes a normalized count matrix as input, which can result from either scRNA-seq or spatial transcriptomics (ST) data. In the latter case, NICHES uses information about the spatial locations of cells or spots as additional input to constrain communication edges.

Additionally, NICHES uses prior knowledge of known ligand-receptor interactions from the FANTOM5 database (Ramilowski et al., 2015) or the OmniPath database (Türei et al., 2021) to construct a CCIM from gene expression data.

The first step in constructing the CCIM is for NICHES to define the edges, i.e. cell pairs, that will be considered when computing ligand-receptor interaction scores. Since considering each possible cell pair is computationally expensive, NICHES limits interaction score computations to a

subset of cell pairs. Specifically, when applied to a count matrix from an scRNA-seq experiment, NICHES requires pre-annotated cell type information to determine which cell pairs are sampled. When spatial information is available, however, NICHES can focus on local spatial neighborhoods, i.e. niches. NICHES can construct three different types of CCIMs: 1) a cell-to-cell interaction matrix where observations correspond to cell pairs; 2) a cell-to-system matrix where, for each cell treated as a sender cell, the receptor expressions are aggregated by computing either the sum or the mean of the receptor expressions in the receiver system; and 3) a system-to-cell matrix where, for each cell treated as a receiver cell, the ligand expressions are aggregated by computing either the sum or the mean of the receptor expressions in the receiver system. Note that a system can have two different meanings depending on whether scRNA-seq data or ST data is used. Given scRNA-seq data, a system of a cell is defined by all cells that are connected to the cell after sub-sampling. For ST data, the system, or neighborhood, i.e., niche of a cell is defined by cells that are spatially close to the cell. Note that all types of CCIMs can be analyzed with the BAE, since the resulting data format is always a matrix.

The second step in constructing the CCIM is the ligand-receptor score computation for each ligand-receptor interaction and each individual cell pair. NICHES computes an interaction score based on the product of the ligand expression in the sender cell and the receptor expression in the receiver cell. To account for multi-subunit ligands and receptors, the final ligand or receptor expression that is used for the product for calculating the interaction score is defined by the product of the individual expressions of the components.

To handle high sparsity levels in the original gene expression matrix, the authors of NICHES recommend using an imputation algorithm, e.g., ALRA (Linderman et al., 2022) prior to applying the NICHES algorithm. Note, that data imputation imputes gene expression values of genes whose expression levels were measured as zeros, where it is not clear weather those imputed values were hidden during measurement or if they are false positives.

S3.3 Preprocessing of the cell-cell interaction matrix

Rarely occurring ligand-receptor interactions and uninformative cell pairs can be filtered from the CCIM prior to applying the BAE for sparse dimensionality reduction. We recommend removing ligand-receptor interactions that have nonzero scores in fewer than 20 cell pairs, as well as cell pairs that exhibit nonzero scores for fewer than 5 distinct ligand-receptor interactions (with the exact thresholds adjustable by the user). This filtering step mitigates the influence of noise that can arise from the amplified sparsity introduced by the multiplicative scoring of ligand and receptor expression, thereby supporting the model to focus on biologically meaningful interaction patterns. After filtering, the remaining features of the CCIM, i.e., the ligand–receptor interaction scores, should be standardized across cell pairs. Specifically, z-scores are computed for each feature vector and used as input for training the BAE model.

S4 Boosting autoencoder scalability analysis

To investigate scalability of our adapted BAE with respect to increasing sample size in single-cell data, we conducted a systematic analysis focused solely on the dimensionality-reduction stage, decoupled from construction of the CCI created with NICHES. We used the 10x Genomics Visium anterior mouse brain data for the scalability analysis (see also Section S6). NICHES was run once on ALRA-imputed data (Linderman et al., 2022) in cell-to-cell spatial mode with $k = 36$

neighbors per spot (capturing 3-hop neighborhoods). After filtering, the CCIM comprised 95442 spot pairs (observations) and 1938 ligand-receptor interactions (features). For the scalability study, we drew random subsamples at 10% increments from 10% to 100% of the CCIM and performed dimensionality reduction for five independent random initializations of the BAE decoder. Model hyperparameters were held fixed across runs: latent dimensionality $= 20$; batch size $= 2^{11}$ (2048); learning rate for decoder updates $= 0.01$; component-wise boosting step size for encoder updates $= 0.01$; one boosting step per training epoch; AdamW weight decay $= 0.1$. Each model was trained for 100 epochs; in every epoch the training data were partitioned into $\lceil n/B \rceil$ mini-batches for n observations and batch size B. We report, as a function of sample size, the sparsity of the initial CCIM, sparsity of the learned BAE encoder weight matrix, wall-clock training time, and total allocated memory (Fig. S1).

In a complementary experiment, we held the sample size fixed and varied the number of features using the same data and hyperparameters (Fig. S2).

Overall, computation time and allocated memory scale linearly with sample size for a fixed number of features. For a fixed sample size, runtime increases near-linearly with the number of features, and memory usage increases linearly. Notably, with fewer features the learned encoder is less sparse after 100 epochs, consistent with a reduced set of selectable features.

Reproducible Jupyter notebooks are available at our GitHub repository: https://github.com/NiklasBrunn/Sparse-dimension-reduction. All experiments were run on a MacBook Pro (2023) with an Apple M2 Max chip (12-core CPU, 38-core GPU) and 96 GB of RAM.

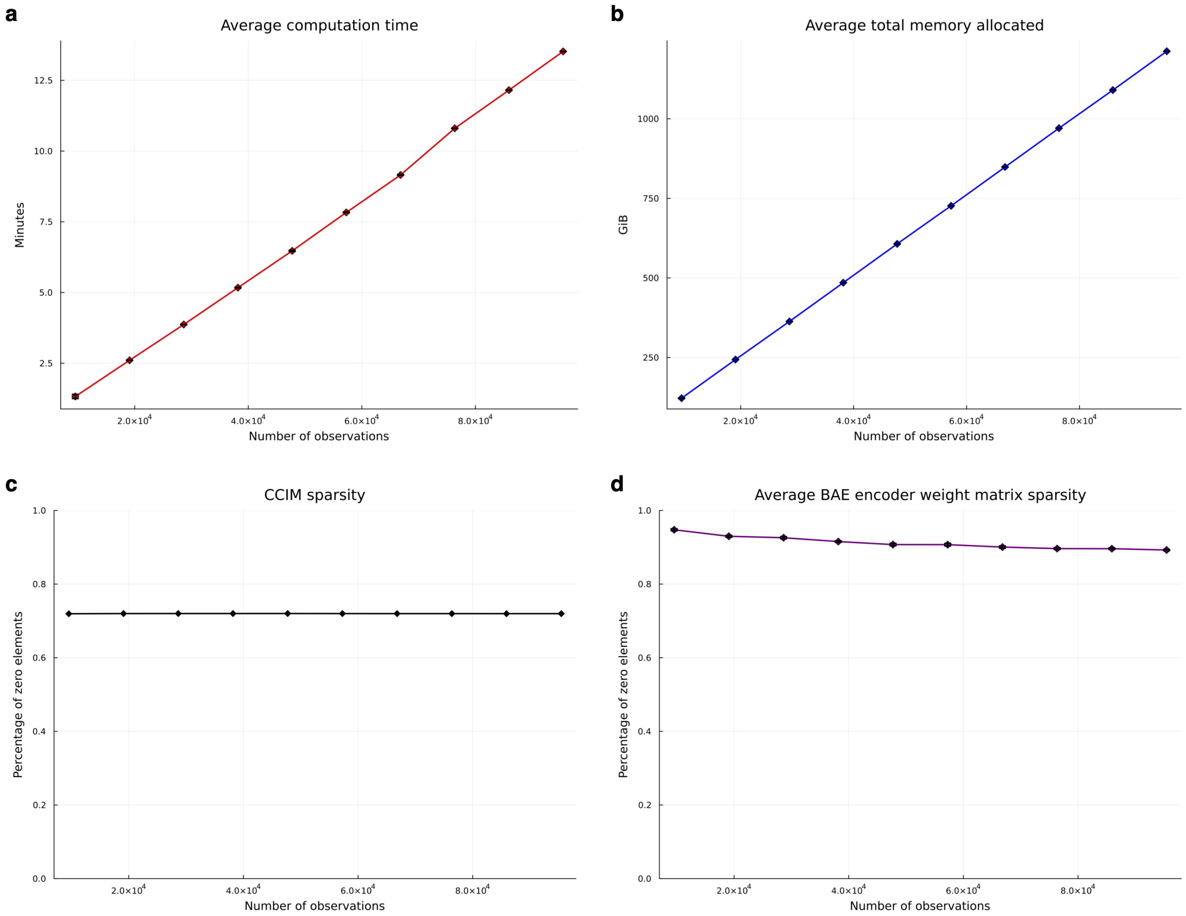

Figure S1: **Scalability analysis results for an increasing number of observations.**

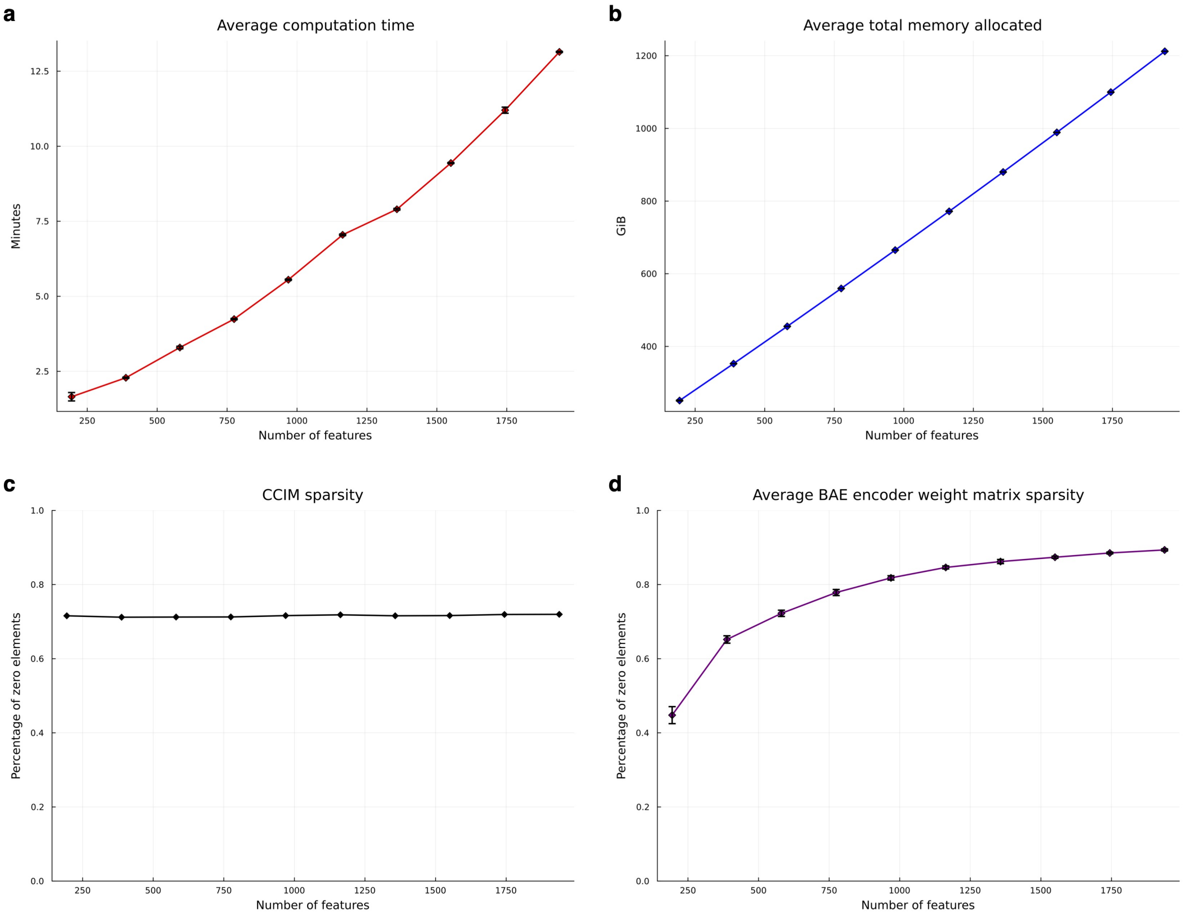

Figure S2: **Scalability analysis results for an increasing number of features.**

S5 Analysis of BAE-identified interactions in rat lung scRNA-seq data

In cluster 26 in Fig. 1 in the main text, the highlighted cell pairs identify fibroblasts as sender cells and interstitial macrophages as receiver cells. In the rat lung, fibroblasts produce the proteoglycan biglycan (BGN), with increased BGN expression and tissue deposition demonstrated during bleomycin-induced fibrosis (Westergren-Thorsson et al., 1993; Koslowski et al., 2001), and xylosyltransferase activity increased in isolated rat lung fibroblasts (Koslowski et al., 2001). Lung macrophage TLR2 expression is injury-responsive in rats, with receptor upregulation demonstrated on alveolar macrophages during ventilator-induced lung injury (Dai et al., 2015). Although the BGN–TLR2 interaction has to our knowledge not been directly investigated in rat lungs, seminal mouse studies established that soluble biglycan activates macrophages through TLR2 and TLR4 in vivo (Schaefer et al., 2005), supporting the plausibility that fibroblast-derived BGN could engage TLR2 on interstitial macrophages in the rat lung.

Further, CSF1R$^+$ lung tissue macrophages (including interstitial macrophages) are present in lung tissue and expand upon CSF-1 stimulation (Irvine et al., 2020). IL-34 is a bona fide ligand of CSF1R (Wang et al., 2012; Lelios et al., 2020) and fibroblasts can produce IL-34 under inflammatory cues in human tissues (Hwang et al., 2012; Boström et al., 2013). In mice, CSF1R inhibition depletes interstitial macrophages and prevents radiation-induced pulmonary fibrosis (Meziani et al., 2018), supporting a model in which fibroblasts act as sender cells (IL-34) and interstitial macrophages as receiver cells (CSF1R) in the rat lung. Although a fibroblast–interstitial macrophage IL-34–CSF1R interaction has not been directly demonstrated in rat lungs, these findings support its plausibility and provide a clear, testable hypothesis.

S6 Analysis of anterior mouse brain spatial transcriptomics data

Our interaction analysis workflow extends naturally to spatial transcriptomics data, operating in cell-to–cell, (sender-)system–to–cell, or cell–to–(receptor-)system modes without requiring preannotated cell/spot labels. Spatial coordinates allow reconstructed cell–cell interactions to be visualized *in situ*, improving interpretability. When coordinates are available, interaction edges can be constrained by plausible diffusion distances for secreted ligands. For 10x Visium data, we restrict edges to the 1-hop neighbors (the six hexagonally adjacent spots, except at tissue borders), corresponding to distances up to approximately $127.5\,\mu\mathrm{m}$ from a spot center (spot diameter $55\,\mu\mathrm{m}$; center-to-center spacing $100\,\mu\mathrm{m}$).

As an example, we analyze an adult anterior mouse brain 10x Visium tissue section provided in our workflow analysis Jupyter notebook. The data can be accessed following the Seurat spatial vignette at https://satijalab.org/seurat/articles/spatial_vignette.html#x-visium. We also provide a download function for this dataset in our GitHub repository. The spatial data with cluster annotations is shown in Fig. S3a.

We performed a spot-label–free system–to–cell analysis to examine how collective sender information acts on individual receiver spots. For each receiver spot, we considered its closest six neighboring sender spots when running NICHES to compute the CCIM, using interactions from the FANTOM5 ligand–receptor database.

Next, we applied structured BAE dimensionality reduction to 1830 ligand–receptor pairs with 20 latent dimensions, yielding 40 possible clusters to which the model can assign niche–cell pairs.

A UMAP of the learned BAE latent space, colored by receiver cluster, is shown in Fig. S3b. The model recapitulates the spot clustering structure of the gene expression data and captures broader spatial tissue organization. For example, clusters 2, 3, 4, 6, and 7, corresponding to cortical layers form an ordered ring-like arrangement. The clustering of the CCIM data induced by the BAE split-softmax representation closely matches the original spot clustering while providing slightly finer resolution (Fig. S3c,d). The encoder weight matrix after training is highly sparse (94.42% zero weights after training), indicating a compact set of model-selected interactions corresponding to each cluster.

The BAE is constrained to learn disentangled latent dimensions. With the split-softmax layer, each latent dimension is further split into two complementary interaction clusters, yielding $2\times$ the number of latent dimensions. Interaction patterns for all clusters are shown in Fig. S4–S6 (each point denotes a niche–to–cell pair). Note that the BAE learns co-expression score patterns from database-defined ligand-receptor pairs. Therefore, even biologically implausible pairs can produce a detectable signal if their genes are co-expressed and results should be interpreted with database curation in mind.

Running NICHES in system-to-spot mode yields latent activity reflecting sender-system influence on receiver spots, with a clustering grouping receivers by their incoming signaling patterns. Within each cluster, linked interactions can be ranked by their nonzero encoder weights and visualized on the tissue for interactions of interest. For clusters 2, 15, 16, and 40, we display the top 10 model-selected ligand–receptor interactions alongside latent cluster activity scores and the system–to–cell interaction scores from the CCIM within tissue for the top-scoring interactions in Fig. S7.

Notably, several of the top 10 ligand–receptor interactions shown in Fig. S7 are known to be functionally active in the anterior mouse brain.

For example, for cluster 2 in Fig. S7a, the receiver-spot hotspot localizes to the ventral olfactory cortex, mainly piriform cortex (PIR) with possible olfactory tubercle, based on Allen Brain Atlas landmarks and the PIR position ventral to the rhinal fissure (Lein et al., 2007; Paxinos et al., 2019). Within this region, two BMP axes from the ranked list of interactions are biologically plausible, BMP7–BMPR1A (ALK3) and BMP5–ACVR1 (ALK2). PIR shows transcript support for *Bmp7* and *Bmpr1a*, and *Acvr1* is broadly expressed across cortex (Lein et al., 2007; Uhlén et al., 2015). BMP type I receptor signaling promotes astroglial differentiation and acts in cortical parenchyma, which supports neuron and astrocyte targets (Mabie et al., 1997; Lein et al., 2007). Chemokine signaling is also credible: *Ccl3* transcripts rise in piriform cortex after seizures (Arisi et al., 2015), CCR5 limits cortical plasticity and is expressed in neurons (Zhou et al., 2016), and CCR1 contributes to central nervous system inflammation (Tian et al., 2024). Finally, F9–LRP1 is feasible at the neurovascular interface because activated factor IXa binds LRP1 and LRP1 is abundant in astrocytes (Neels et al., 2000; Liu et al., 2017).

In Fig. S7b, the highlighted brain region for cluster 15 aligns with the caudoputamen (CP) based on Allen Brain Atlas and Paxinos landmarks (Lein et al., 2007; Paxinos et al., 2019). Among the BAE's top selected interactions, two have strong support in CP. EFNB2–EPHA4 fits known Eph/ephrin signaling in striatum, which marks matrix/striosome patterning and shapes striatal circuitry, and structural work shows that EphA4 can bind ephrin-B2 (Janis et al., 1999; Passante et al., 2008; Qin et al., 2010). TGF-α–EGFR is plausible for astrocyte-mediated signaling in CP, since TGF-α is expressed in striatum and activates EGFR to drive astrogliosis in adult CNS (Weickert et al., 1995; Rabchevsky et al., 1998).

For cluster 16 in Fig. S7c, the receiver-spot activation pattern follows the neocortical surface

and matches Layer 2/3 (L2/3) based on Allen Brain Atlas and Paxinos landmarks (Lein et al., 2007; Paxinos et al., 2019). Among the BAE top-ranked interactions, several are biologically plausible for L2/3. VIP–VPAC1 fits the dense VIP circuitry in L2/3 and VPAC1 pharmacology (Garrett et al., 2020; Harmar et al., 2012). HDC–HRH3 is consistent with the widespread presynaptic H_3 receptor in cortex that modulates transmitter release (Schlicker et al., 2016). CCL8–CCR2 is credible given CCR2's bona fide ligands (including CCL8) operating at cortical perivascular/meningeal interfaces captured by superficial spots (She et al., 2022; Silvin et al., 2023). Finally, HGF–SDC2 is plausible because syndecan-2 is enriched at dendritic spines and heparan-sulfate syndecans can present HGF to MET to potentiate signaling (Ethell et al., 1999; Derksen et al., 2002; Sarrazin et al., 2011).

For cluster 40 in Fig. S7d, the receiver-spot band sits deeper than the L2/3 ribbon and best matches the Layer 4 and Layer 5 region (Lein et al., 2007; Paxinos et al., 2019). Among the BAE top-ranked ligand–receptor interactions, the most plausible in this cortical region include integrin-centered adhesion signaling (ADAM23–ITGB3 and ADAM15–ITGB3), consistent with ADAM23 modulating $\alpha_v\beta_3$ activation and ADAM15 binding $\alpha_v\beta_3$ via its RGD motif at cell–cell/ECM contacts relevant to synaptic adhesion (Verbisck et al., 2009; Krätzschmar et al., 1996; Nath et al., 1999). Synaptogenic cues are also well supported: FGF22–FGFR2, in line with FGF22 acting as a presynaptic organizer through FGFRs (Umemori et al., 2004), and SLIT2–ROBO2, consistent with ROBO2-driven synapse specification in excitatory pathways (Blockus et al., 2021; Brose et al., 1999).

Together, these results demonstrate that our cell–cell interaction analysis workflow recapitulates mechanistically credible ligand–receptor interactions and yields localized, testable hypotheses about region-specific signaling for future experimental validation.

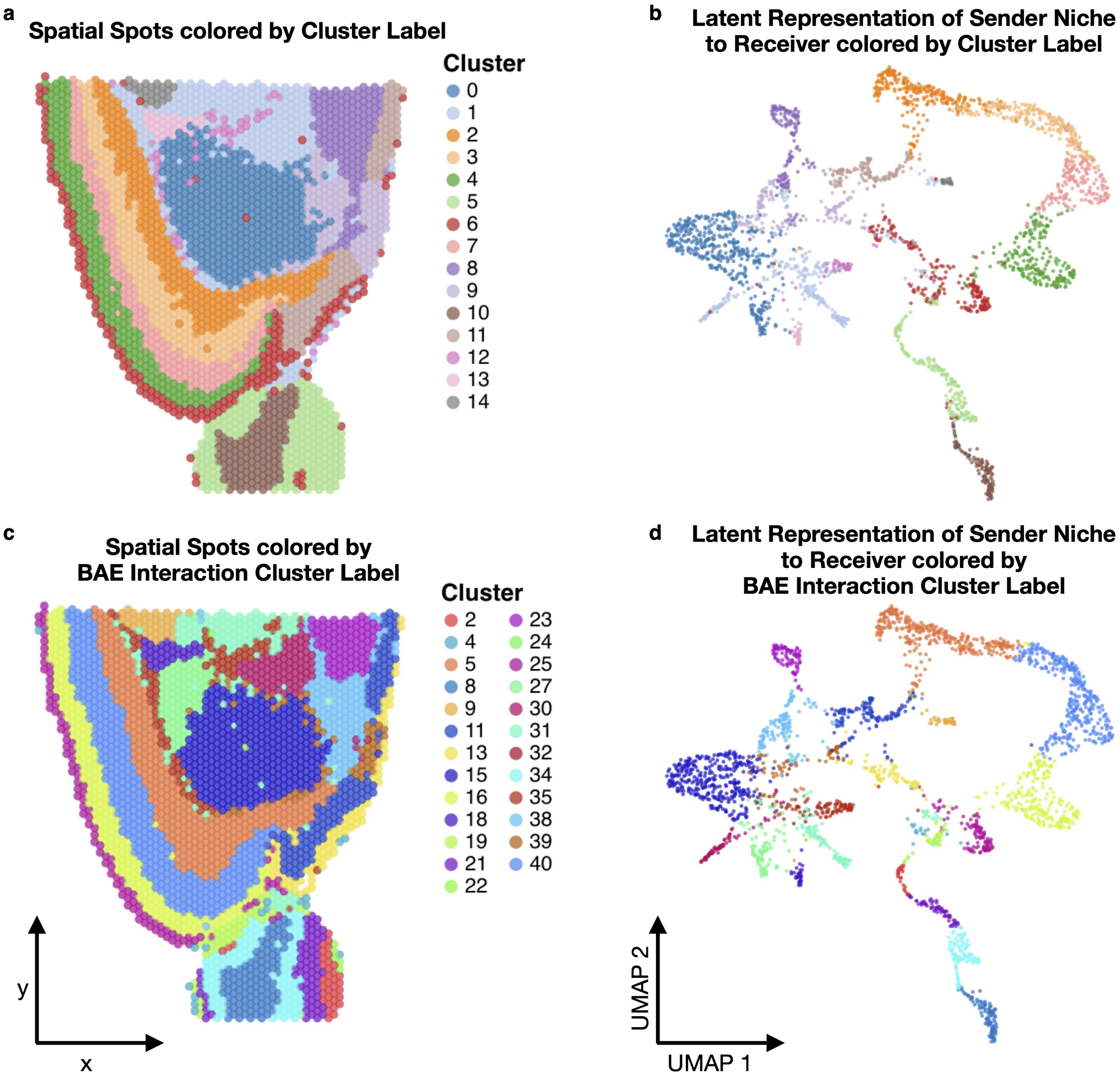

Figure S3: **Clustering results visualized in tissue and BAE latent space.** **a** Spatial map of spots colored by clusters derived from gene expression profiles. **b** UMAP of the BAE latent representation of the CCIM data, colored by the same spot clusters. **c** Spatial map of spots colored by BAE-derived system-to-receiver spot pair clustering results based on CCIM data. Cluster labels are shown only for clusters with at least one assigned system-to-receiver spot pair. **d** UMAP of the BAE latent representation of the CCIM data, colored by the same BAE-derived system-to-receiver spot pair clusters.

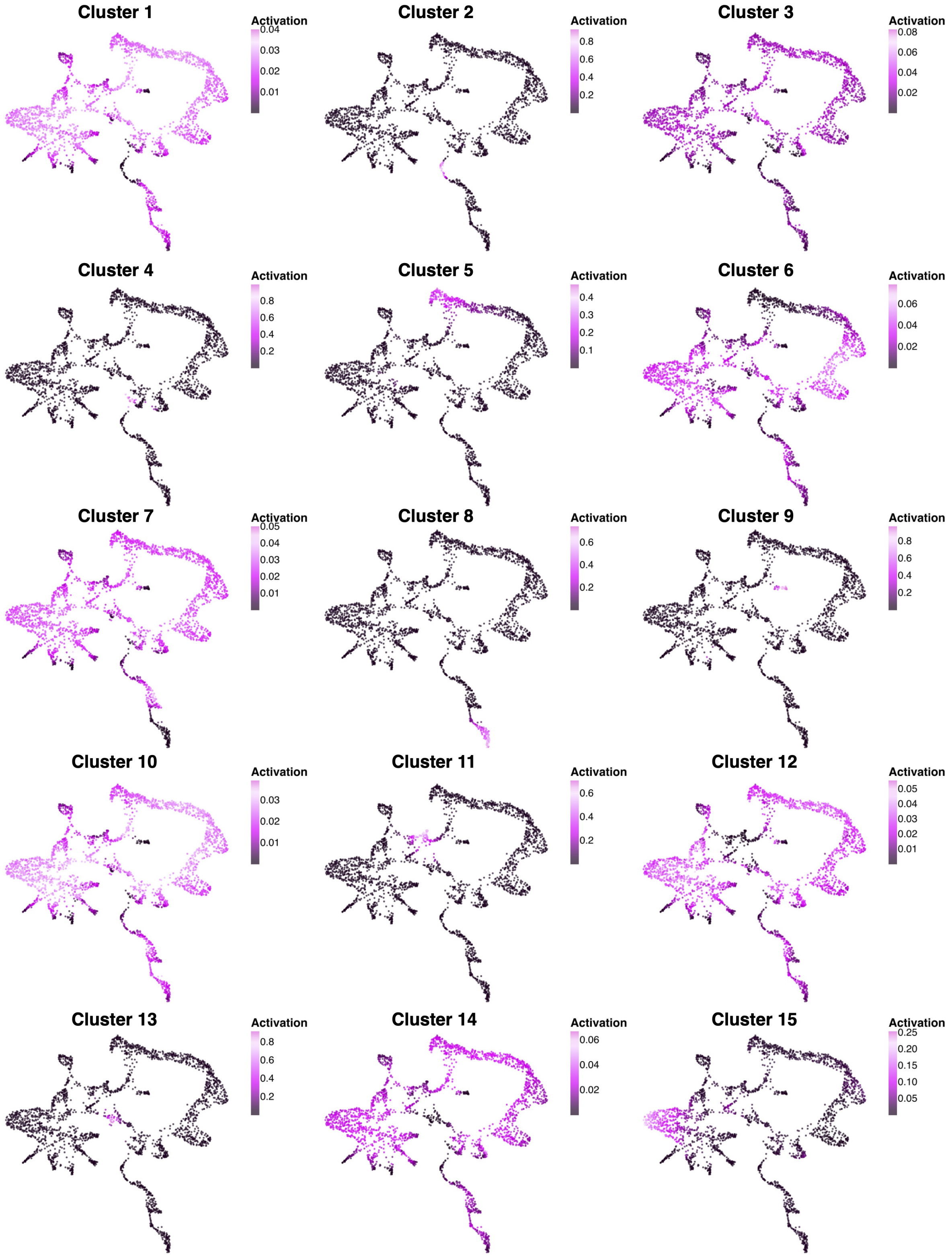

Figure S4: **UMAP of the BAE latent space.** Points represent system–to–receiver spot pairs. Color encodes split-softmax activation score (clusters 1–15 are shown).

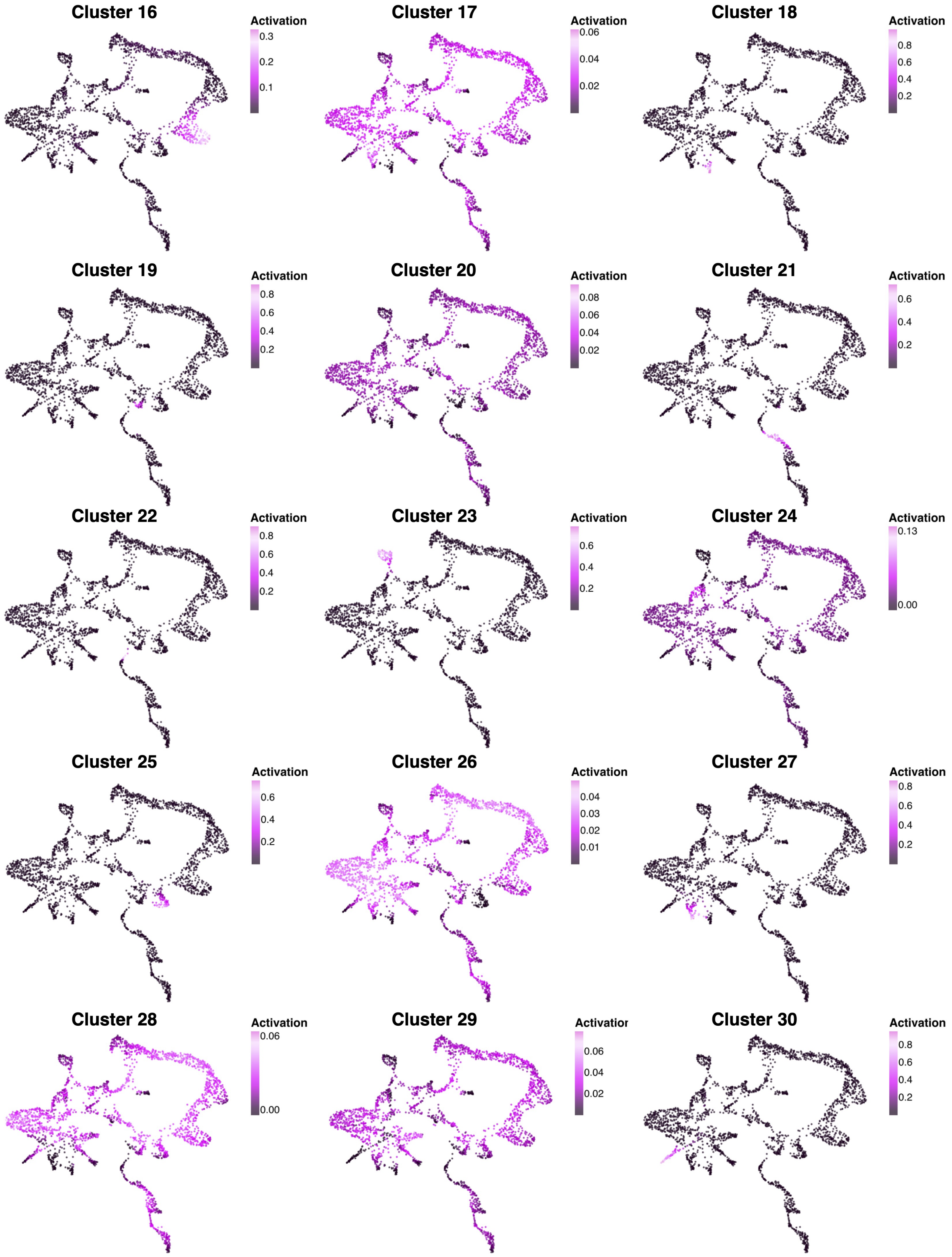

Figure S5: **UMAP of the BAE latent space.** Points represent system–to–receiver spot pairs. Color encodes split-softmax activation score (clusters 16–30 are shown).

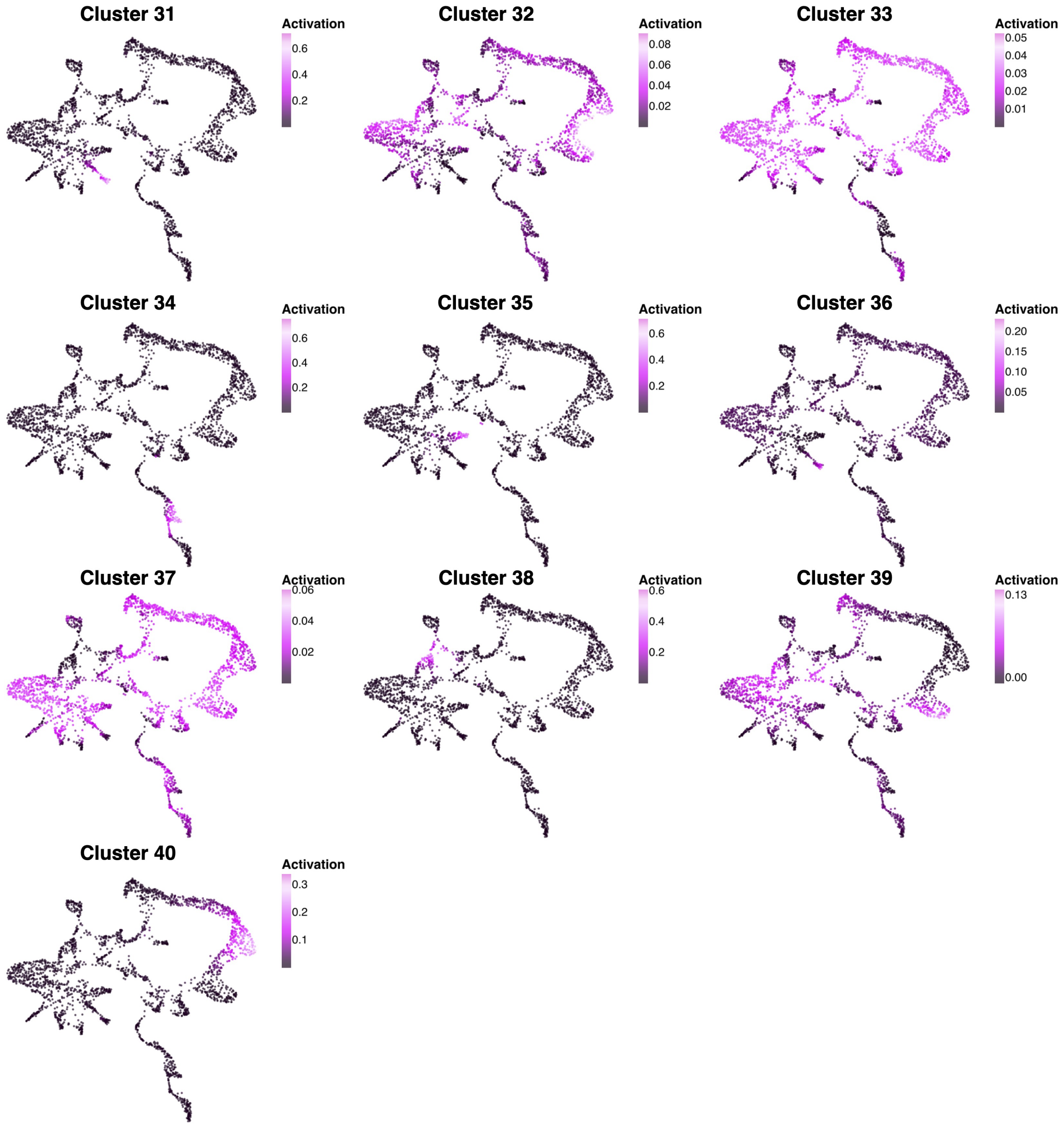

Figure S6: **UMAP of the BAE latent space.** Points represent system–to–receiver spot pairs. Color encodes split-softmax activation score (clusters 31–40 are shown).

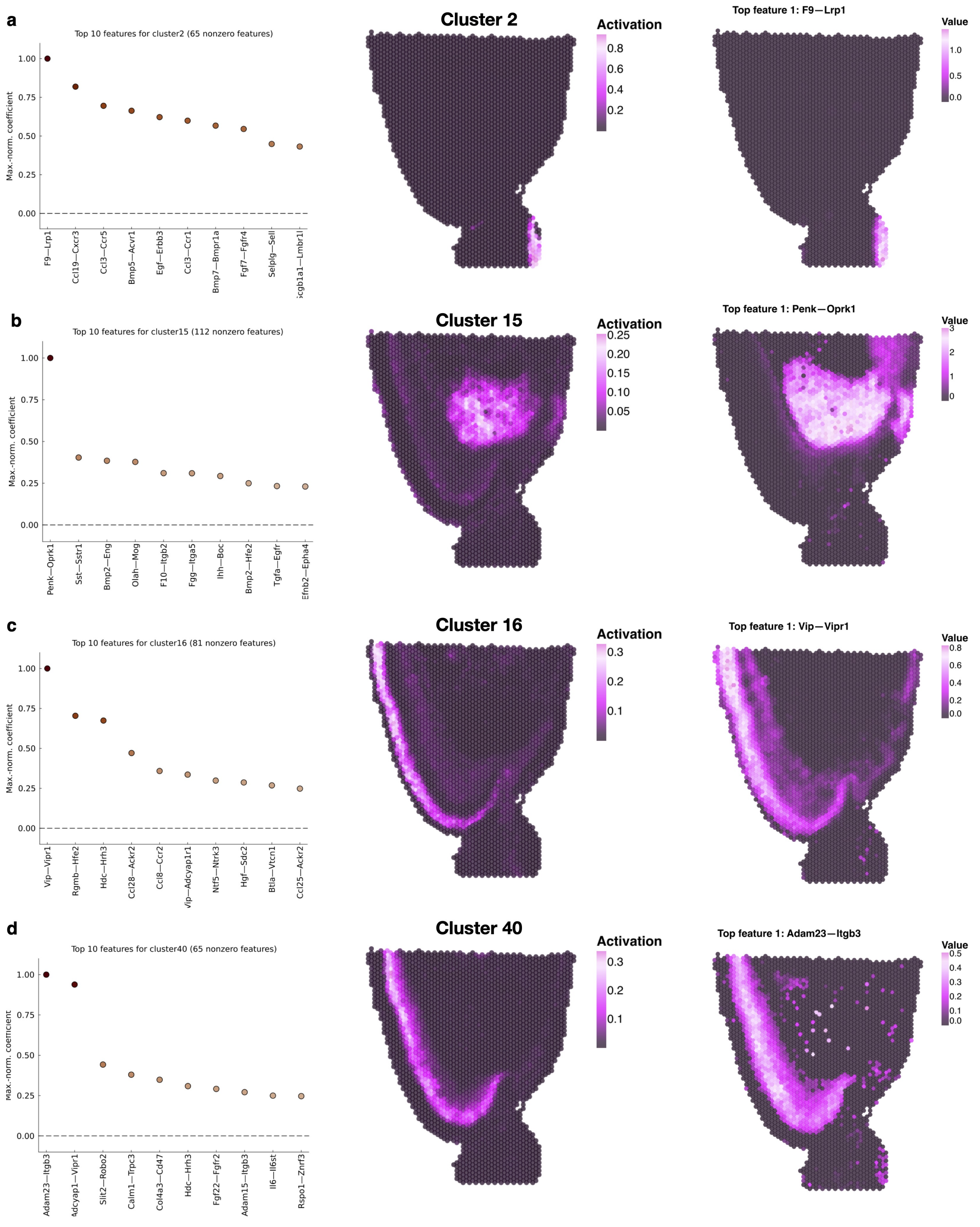

Figure S7: **Spatial BAE cluster analysis.** For cluster 2 (**a**), 15 (**b**), 16 (**c**), 40 (**d**) results, from left to right: Top 10 ranked BAE-selected ligand–receptor interactions, latent softmax activation scores mapped in tissue, and NICHES-derived system-to-receiver spot pair interaction scores in tissue.

S7 References

Andrews TS and Hemberg M. Identifying cell populations with scRNASeq. Molecular aspects of medicine 2018;59:114–22.

Arisi GM, Foresti ML, Katki K, and Shapiro LA. Increased CCL2, CCL3, CCL5, and IL-1β cytokine concentration in piriform cortex, hippocampus, and neocortex after pilocarpine-induced seizures. Journal of neuroinflammation 2015;12:129.

Bezanson J, Edelman A, Karpinski S, and Shah VB. Julia: A Fresh Approach to Numerical Computing. SIAM Review 2017;59:65–98.

Blockus H, Rolotti SV, Szoboszlay M, Peze-Heidsieck E, Ming T, Schroeder A, Apostolo N, Vennekens KM, Katsamba PS, Bahna F, et al. Synaptogenic activity of the axon guidance molecule Robo2 underlies hippocampal circuit function. Cell reports 2021;37.

Boström EA and Lundberg P. The newly discovered cytokine IL-34 is expressed in gingival fibroblasts, shows enhanced expression by pro-inflammatory cytokines, and stimulates osteoclast differentiation. PloS one 2013;8:e81665.

Brose K, Bland KS, Wang KH, Arnott D, Henzel W, Goodman CS, Tessier-Lavigne M, and Kidd T. Slit proteins bind Robo receptors and have an evolutionarily conserved role in repulsive axon guidance. Cell 1999;96:795–806.

Dai H, Pan L, Lin F, Ge W, Li W, and He S. Mechanical ventilation modulates Toll-like receptors 2, 4, and 9 on alveolar macrophages in a ventilator-induced lung injury model. Journal of thoracic disease 2015;7:616.

Derksen PW, Keehnen RM, Evers LM, Van Oers MH, Spaargaren M, and Pals ST. Cell surface proteoglycan syndecan-1 mediates hepatocyte growth factor binding and promotes Met signaling in multiple myeloma. Blood, The Journal of the American Society of Hematology 2002;99:1405–10.

Ethell IM and Yamaguchi Y. Cell surface heparan sulfate proteoglycan syndecan-2 induces the maturation of dendritic spines in rat hippocampal neurons. The Journal of cell biology 1999;144:575–86.

Garrett M, Manavi S, Roll K, Ollerenshaw DR, Groblewski PA, Ponvert ND, Kiggins JT, Casal L, Mace K, Williford A, et al. Experience shapes activity dynamics and stimulus coding of VIP inhibitory cells. elife 2020;9:e50340.

Glorot X and Bengio Y. Understanding the difficulty of training deep feedforward neural networks. In: *Proceedings of the thirteenth international conference on artificial intelligence and statistics*. JMLR Workshop and Conference Proceedings. 2010:249–56.

Hackenberg M, Brunn N, Vogel T, and Binder H. Infusing structural assumptions into dimensionality reduction for single-cell RNA sequencing data to identify small gene sets. Communications Biology 2025;8:414.

Harmar AJ, Fahrenkrug J, Gozes I, Laburthe M, May V, Pisegna JR, Vaudry D, Vaudry H, Waschek JA, and Said SI. Pharmacology and functions of receptors for vasoactive intestinal peptide and pituitary adenylate cyclase-activating polypeptide: IUPHAR review 1. British journal of pharmacology 2012;166:4–17.

Hess M, Hackenberg M, and Binder H. Exploring generative deep learning for omics data using log-linear models. Bioinformatics 2020;36:5045–53.

Hwang SJ, Choi B, Kang SS, Chang JH, Kim YG, Chung YH, Sohn DH, So MW, Lee CK, Robinson WH, et al. Interleukin-34 produced by human fibroblast-like synovial cells in rheumatoid arthritis supports osteoclastogenesis. Arthritis research & therapy 2012;14:R14.

Innes M. Flux: Elegant machine learning with Julia. Journal of Open Source Software 2018;3:602.

Innes M, Edelman A, Fischer K, Rackauckas C, Saba E, Shah VB, and Tebbutt W. A differentiable programming system to bridge machine learning and scientific computing. arXiv preprint arXiv:1907.07587 2019.

Irvine KM, Caruso M, Cestari MF, Davis GM, Keshvari S, Sehgal A, Pridans C, and Hume DA. Analysis of the impact of CSF-1 administration in adult rats using a novel Csf1r-mApple reporter gene. Journal of leukocyte biology 2020;107:221–35.

Janis LS, Cassidy RM, and Kromer LF. Ephrin-A binding and EphA receptor expression delineate the matrix compartment of the striatum. Journal of Neuroscience 1999;19:4962–71.

Kingma DP and Ba J. Adam: A method for stochastic optimization. arXiv preprint arXiv:1412.6980 2014.

Koslowski R, Pfeil U, Fehrenbach H, Kasper M, Skutelsky E, and Wenzel K. Changes in xylosyltransferase activity and in proteoglycan deposition in bleomycin-induced lung injury in rat. European Respiratory Journal 2001;18:347–56.

Krätzschmar J, Lum L, and Blobel CP. Metargidin, a Membrane-anchored Metalloprotease–Disintegrin Protein with an RGD Integrin Binding Sequence. Journal of Biological Chemistry 1996;271:4593–6.

Lein ES, Hawrylycz MJ, Ao N, Ayres M, Bensinger A, Bernard A, Boe AF, Boguski MS, Brockway KS, Byrnes EJ, et al. Genome-wide atlas of gene expression in the adult mouse brain. Nature 2007;445:168–76.

Lelios I, Cansever D, Utz SG, Mildenberger W, Stifter SA, and Greter M. Emerging roles of IL-34 in health and disease. Journal of Experimental Medicine 2020;217:e20190290.

Linderman GC, Zhao J, Roulis M, Bielecki P, Flavell RA, Nadler B, and Kluger Y. Zero-preserving imputation of single-cell RNA-seq data. Nature communications 2022;13:192.

Liu CC, Hu J, Zhao N, Wang J, Wang N, Cirrito JR, Kanekiyo T, Holtzman DM, and Bu G. Astrocytic LRP1 mediates brain Aβ clearance and impacts amyloid deposition. Journal of Neuroscience 2017;37:4023–31.

Loshchilov I and Hutter F. Decoupled weight decay regularization. arXiv preprint arXiv:1711.05101 2017.

Luecken MD and Theis FJ. Current best practices in single-cell RNA-seq analysis: a tutorial. Molecular systems biology 2019;15:e8746.

Mabie P, Mehler M, Marmur R, Papavasiliou A, Song Q, and Kessler J. Bone morphogenetic proteins induce astroglial differentiation of oligodendroglial–astroglial progenitor cells. Journal of Neuroscience 1997;17:4112–20.

Meziani L, Mondini M, Petit B, Boissonnas A, Montpreville VT de, Mercier O, Vozenin MC, and Deutsch E. CSF1R inhibition prevents radiation pulmonary fibrosis by depletion of interstitial macrophages. European Respiratory Journal 2018;51.

Nath D, Slocombe PM, Stephens PE, Warn A, Hutchinson GR, Yamada KM, Docherty AJ, and Murphy G. Interaction of metargidin (ADAM-15) with αvβ3 and α5β1 integrins on different haemopoietic cells. Journal of cell science 1999;112:579–87.

Neels JG, Berg BM van den, Mertens K, Maat H ter, Pannekoek H, Zonneveld AJ van, and Lenting PJ. Activation of factor IX zymogen results in exposure of a binding site for low-density lipoprotein receptor–related protein. Blood, The Journal of the American Society of Hematology 2000;96:3459–65.

Passante L, Gaspard N, Degraeve M, Frisén J, Kullander K, De Maertelaer V, and Vanderhaeghen P. Temporal regulation of ephrin/Eph signalling is required for the spatial patterning of the mammalian striatum. 2008.

Paxinos G and Franklin KB. Paxinos and Franklin's the mouse brain in stereotaxic coordinates. Academic press, 2019.

Qin H, Noberini R, Huan X, Shi J, Pasquale EB, and Song J. Structural characterization of the EphA4-Ephrin-B2 complex reveals new features enabling Eph-ephrin binding promiscuity. Journal of Biological Chemistry 2010;285:644–54.

Rabchevsky AG, Weinitz JM, Coulpier M, Fages C, Tinel M, and Junier MP. A role for transforming growth factor α as an inducer of astrogliosis. Journal of Neuroscience 1998;18:10541–52.

Ramilowski JA, Goldberg T, Harshbarger J, Kloppmann E, Lizio M, Satagopam VP, Itoh M, Kawaji H, Carninci P, Rost B, et al. A draft network of ligand–receptor-mediated multicellular signalling in human. Nature communications 2015;6:7866.

Raredon MSB, Adams TS, Suhail Y, Schupp JC, Poli S, Neumark N, Leiby KL, Greaney AM, Yuan Y, Horien C, et al. Single-cell connectomic analysis of adult mammalian lungs. Science advances 2019;5:eaaw3851.

Raredon MSB, Yang J, Kothapalli N, Lewis W, Kaminski N, Niklason LE, and Kluger Y. Comprehensive visualization of cell–cell interactions in single-cell and spatial transcriptomics with NICHES. Bioinformatics 2023;39:btac775.

Sarrazin S, Lamanna WC, and Esko JD. Heparan sulfate proteoglycans. Cold Spring Harbor perspectives in biology 2011;3:a004952.

Schaefer L, Babelova A, Kiss E, Hausser HJ, Baliova M, Krzyzankova M, Marsche G, Young MF, Mihalik D, Götte M, et al. The matrix component biglycan is proinflammatory and signals through Toll-like receptors 4 and 2 in macrophages. The Journal of clinical investigation 2005;115:2223–33.

Schlicker E and Kathmann M. Role of the histamine H3 receptor in the central nervous system. Histamine and histamine receptors in health and disease 2016:277–99.

She S, Ren L, Chen P, Wang M, Chen D, Wang Y, and Chen H. Functional roles of chemokine receptor CCR2 and its ligands in liver disease. Frontiers in immunology 2022;13:812431.

Silvin A, Qian J, and Ginhoux F. Brain macrophage development, diversity and dysregulation in health and disease. Cellular & molecular immunology 2023;20:1277–89.

Tian Q, Yan Z, Guo Y, Chen Z, and Li M. Inflammatory role of CCR1 in the central nervous system. Neuroimmunomodulation 2024;31:173–82.

Türei D, Valdeolivas A, Gul L, Palacio-Escat N, Klein M, Ivanova O, Ölbei M, Gábor A, Theis F, Módos D, et al. Integrated intra-and intercellular signaling knowledge for multicellular omics analysis. Molecular systems biology 2021;17:e9923.

Uhlén M, Fagerberg L, Hallström BM, Lindskog C, Oksvold P, Mardinoglu A, Sivertsson Å, Kampf C, Sjöstedt E, Asplund A, et al. Tissue-based map of the human proteome. Science 2015;347:1260419.

Umemori H, Linhoff MW, Ornitz DM, and Sanes JR. FGF22 and its close relatives are presynaptic organizing molecules in the mammalian brain. Cell 2004;118:257–70.

Verbisck NV, Costa ET, Costa FF, Cavalher FP, Costa MD, Muras A, Paixão VA, Moura R, Granato MF, Ierardi DF, et al. ADAM23 negatively modulates αvβ3 integrin activation during metastasis. Cancer research 2009;69:5546–52.

Wang Y, Szretter KJ, Vermi W, Gilfillan S, Rossini C, Cella M, Barrow AD, Diamond MS, and Colonna M. IL-34 is a tissue-restricted ligand of CSF1R required for the development of Langerhans cells and microglia. Nature immunology 2012;13:753–60.

Weickert CS and Blum M. Striatal TGF-α: postnatal developmental expression and evidence for a role in the proliferation of subependymal cells. Developmental brain research 1995;86:203–16.

Westergren-Thorsson G, Hernnäs J, Särnstrand B, Oldberg Å, Heinegård D, Malmström A, et al. Altered expression of small proteoglycans, collagen, and transforming growth factor-beta 1 in developing bleomycin-induced pulmonary fibrosis in rats. The Journal of clinical investigation 1993;92:632–7.

Wilk AJ, Shalek AK, Holmes S, and Blish CA. Comparative analysis of cell–cell communication at single-cell resolution. Nature Biotechnology 2024;42:470–83.

Zhou M, Greenhill S, Huang S, Silva TK, Sano Y, Wu S, Cai Y, Nagaoka Y, Sehgal M, Cai DJ, et al. CCR5 is a suppressor for cortical plasticity and hippocampal learning and memory. elife 2016;5:e20985.
